# Transmigration of a retained surgical sponge: a case report

**DOI:** 10.1186/s13037-018-0168-y

**Published:** 2018-08-11

**Authors:** Tushar Patial, Namit Rathore, Angesh Thakur, Digvijay Thakur, Kanika Sharma

**Affiliations:** 1 The Clinic, Sanjauli, Shimla, Himachal Pradesh 171006 India; 2 Department of General Surgery, Indira Gandhi Medical College, Shimla, Himachal Pradesh India; 3 Department of Urology, Post Graduate Institute of Medical Education and Research, Chandigarh, India; 4 Department of Radiation Therapy, Rajiv Gandhi Cancer Hospital and Research Centre, New Delhi, India

**Keywords:** Gossypiboma, Textiloma, Retained surgical items, Retained surgical sponge, Case report

## Abstract

**Background:**

A retained surgical sponge remains a dreaded complication of modern surgery. Despite the increasing focus on patient safety instances of “a sponge being left in the abdomen”, are all too common in popular media. In this article we report the rare phenomenon of transmigration of a retained surgical sponge in a patient who underwent laparoscopic sterilization.

**Case presentation:**

A 30-year-old female presented with progressive abdominal pain for about one month and vomiting with obstipation for 2 days. The patient had undergone laparoscopic sterilization 7 years back and then underwent re-canalization one year back. She underwent an exploratory laparotomy for suspected adhesive small bowel obstruction. During surgery, an intra-luminal surgical sponge was recovered from the distal small bowel. The patient recovered and was discharged in good health.

**Conclusion:**

Despite numerous advances in terms of technology and the ever-growing emphasis on patient safety, the problem of a retained surgical sponge remains a dreaded potential complication. All clinicians and health care professionals should be aware of this entity and its various presentations.

## Background

All over the world over 312.9 million major operations are carried out each year [1]. Retained foreign bodies (RFB) are an undesirable and avoidable complication of surgery. In literature, the incidence of RFB is 0.01 to 0.001% and in 80% of these events the offender is a surgical sponge [2]. Reports of the condition merely the scratch the surface as there is a discrepancy between the number of surgeries performed and the documented frequency of retained surgical sponges. This may be due to under reporting, possible legal ramifications and the reputation of hospital and the doctors involved. The condition has been described after virtually every kind of surgery - abdominal, thoracic and head and neck with disastrous consequences for patients as well as for doctors [3, 4]. Herein we present such a case with the rare event of transmigration of the retained surgical sponge.

## Case report

A 30-year-old female presented to the emergency with progressive abdominal pain for about one month, which had increased in severity for the past 2 days. The pain was initially localized to the right iliac fossa, and was described as colicky, lasting for about 4–5 min with 2–3 episodes/day, partially relieved by analgesics. Over the past 2 days, the severity of the pain had increased and had become generalized. She also had multiple episodes of severe vomiting accompanied by obstipation for the same amount of time.

**Fig. 1:**
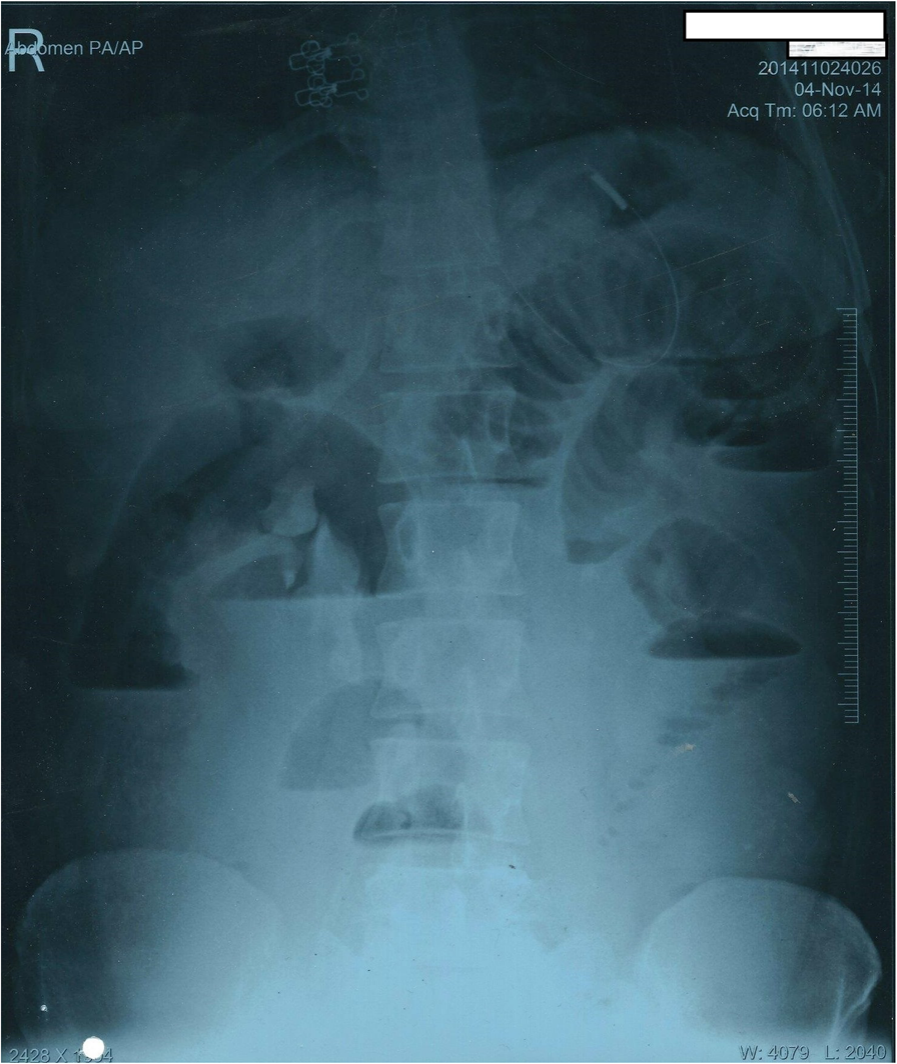
Abdominal radiograph erect showing, multiple air-fluid levels

**Fig. 2:**
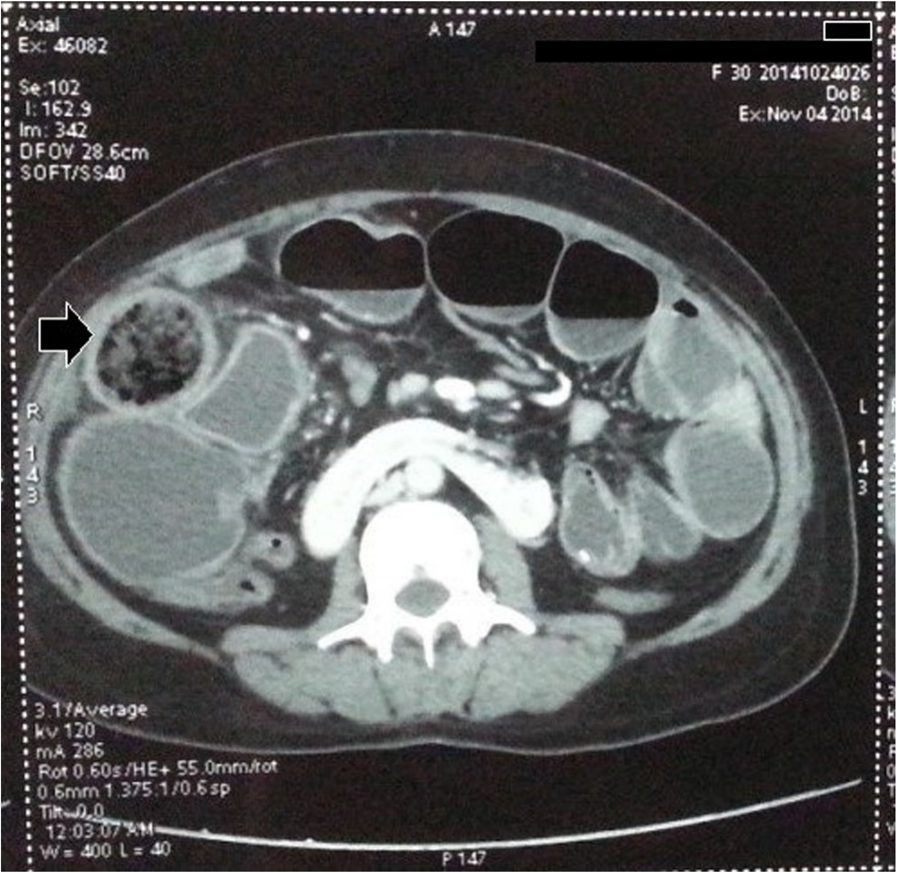
Computed tomography of the abdomen, showing intraluminal gossypiboma (Black arrow)

**Fig. 3:**
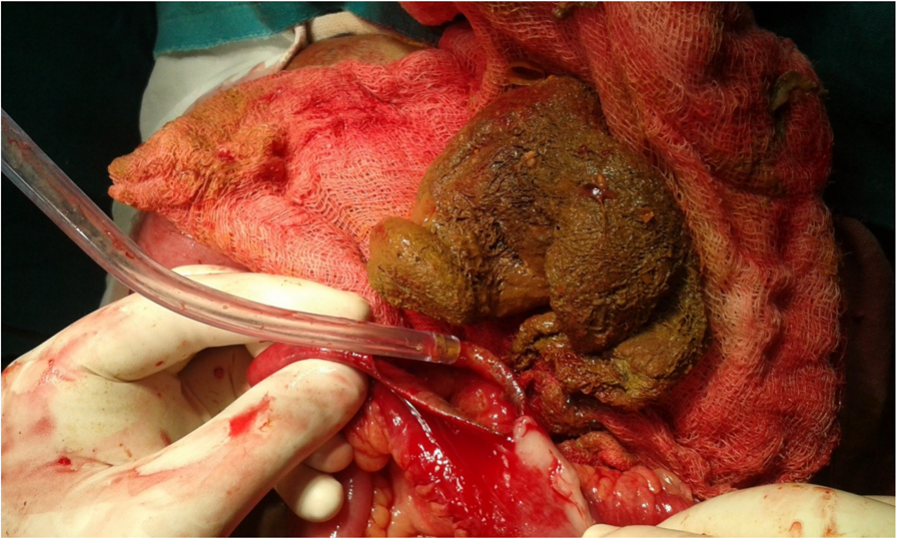
Intra operative photograph, after retrieval of gossypiboma

The patient had undergone laparoscopic sterilization 7 years ago and then underwent re-canalization one year back. The patient was initially managed at a primary health centre and was then referred to our hospital with a tentative diagnosis of small bowel obstruction due to adhesions with worsening of symptoms. On examination, the patient had tachycardia, abdominal distension with guarding and rigidity. No abdominal mass was palpable. A per rectal examination revealed hard fecal matter with rectal ballooning. A plain erect abdominal radiograph revealed multiple air-fluid levels suggestive of small bowel obstruction (Fig. 1). Sonology was non-contributory. An abdominal computed tomography scan was suggestive of a mass lesion in the small intestine with mottled appearance (Fig. 2). On surgical exploration, the small bowel was distended till about 30 cm from the ileocecal junction. An enterotomy was made at the site of the palpable sponge and the sponge was retrieved (Fig. 3). Post operatively, the patient developed surgical site infection and was eventually discharged on day 7 of admission.

## Discussion

A retained surgical sponge is often referred to as a “Gossypiboma”. The word is derived from ‘Gossypium’, which is Latin for cotton, and ‘oma’ which is Greek for tumor/mass. Other terms with similar meanings include, “Gauzeom”, “Textiloma”, “Cottonoma” and “Muslinoma” [5–7]. Based on the source referenced, the incidence of the condition varies between 1/100 and 1/3000 for all surgical interventions and from 1/1000 to 1/1500 for intra-abdominal operations [8].

Transmigration of the surgical sponge a fascinatingly rare event. A 2008 study published by Zandvoort et al. found a total of 64 cases reported worldwide [9]. A subsequent study from 2017 found transmigration present in 36 cases, reported exclusively from India [10].

In vivo studies in dogs have revealed that the body can respond in one of two ways with respect to gossypibomas. The antigenic potential of the offending agent and the degree of inflammatory response elicited within an individual determines the nature of response [11].

An exudative response is supported by a higher antigenicity and/or a severe inflammatory reaction. This response occurs early and begins abscess formation around the gossypiboma. Increasing pressure leads to breach of the weaker wall, which oftentimes is the gut, resulting in fistula formation and ultimately driving the surgical sponge into the lumen of gut. Peristaltic waves may further augment the deliverance of the gossypiboma into the lumen. This migration of the gossypiboma from the abdomen downregulates the inflammatory response at the site and the process of healing begins by stimulation of fibroblasts. This can often fillup the tract with or without a scar, hence leaving no proof of gut wall perforation. Once inside the lumen, the sponge is thrust onward by peristalsis. If small the sponge may pass spontaneously in stool or it may get stuck at narrow portions of the intestinal lumen. In cases of partial intestinal obstruction, undigested food residue accumulates adjacent to the site of blockage, followed by a few episodes of partial obstruction before becoming complete [10, 11].

If the abscess abuts the abdominal wall it may appear as a septic mass and subsequent rupture of this mass can result in fistulisation as well. Close proximity to the diaphragm can lead to rupture and migration into the thoracic cavity [10, 11].

On the other hand, the transudative response is favoured by low level of antigenicity and/or milder inflammatory response and usually occurs late. This can result in encapsulation of the sponge, resulting in the formation of a mass, or may result in the formation of adhesions, calcification, degradation and uncommonly migration of the gossypiboma as in the exudative response. In contrast to the tissue which surrounds foreign material at other anatomical sites, the tissue encapsulating foreign objects in the peritoneal cavity is avascular in nature. The presence of cytokines may produce anorexia, weakness, weight loss and fever [9, 10].

Gossypibomas may remain asymptomatic for long periods of time depending upon the size, site, and the inflammatory response of body. It may also present with vague ill health, weight loss, fever with chills and rigors, altered bowel habits, anorexia, nausea, vomiting, tenesmus, diarrhoea, discharging sinus, non-healing wound, intestinal obstruction, malabsorption, and as an abdominal mass [12]. The abdominal mass may present as an abscess, or as a pseudotumour [10]. A mass lesion may be confused with location specific common masses like a tubo-ovarian mass or even a hydatid cyst of the liver in right hypochondrium. Cases of spontaneous expulsion have also been reported in literature [13].

Various risk factors have been described in literature. Emergency surgery, prolonged surgery, unplanned change in the surgical procedure, multiple operating teams, change of members of operating team, obesity, female gender, inexperienced staff, improper counting of surgical towels, sticking of towels, small sponges, haemodynamic instability, and poor communication amongst the surgical team have all been noted in reported cases/series [14, 15]. Radiological evaluation plays an important role in the diagnosis. Radiopaque thread impregnated surgical sponges were introduced in 1929 by Cahn and came into general use in the US by the 1940’s. However, it must be remembered that the radiopaque marker may be lost due to twisting or folding of the sponge upon itself [16, 17].

On sonography the sponge is often described as a well-defined mass with linear or wavy internal echogenic area with intense posterior acoustic shadowing. Computed tomography is the investigation of choice, with detection of the sponge as a mass with a well-defined capsule. On occasion, it may have a spongiform or mottled appearance due to air bubbles and rarely even barium may be seen inside sponge [16]. On magnetic resonance imaging, the gossypiboma appears as a well-defined mass with low intensity peripheral wall on T1 &T2 weighted images accompanied by, peripheral wall enhancement and central stripes on gadolinium enhancement on T1 weighted imaging [2, 16].

Once diagnosed, the only treatment of a gossypiboma is its removal [10]. There are many techniques available to prevent the problem of a gossypiboma. The association of registered nurses, recommends that counts should be performed at various phases during surgery. This includes a count performed prior to the start of any procedure, at the time of addition of a new item, prior to closure of a cavity within a cavity, at the time of closure of incision and at skin closure. If any discrepancy is found, it is the duty of the entire surgical team to look for the missing item [18]. If, however, the item cannot be located a radiograph should be taken before the closure [19]. The American college of surgeons endorses the same view and emphasizes that the optimal environment inside the operating room should allow for focussed performance of operative tasks [20].

Bar code based, or data matrix coded sponges have also been used as an adjunct to increase the accuracy of a sponge count [21]. However, both methods require greater time and have increased cost [22]. Radiofrequency scanning has also been evaluated and consists of a wand which can scan a microchip present in the sponge when waved over it. Although this system is superior to standard radiographs, the possibility of missing a sponge still exists if the wand is not used properly or if the microchip sponge is not used [23].

Although it is easy to assume that the fault lies with one individual, evidence suggests that most cases of a retained surgical item are because of team/system errors [24]. Reason’s model of accident causation suggests that organisations which operate in potentially harmful environments, have defences in place that can be brought down by active or latent factors. Latent factors, such as inadequate staffing may not be harmful in themselves, but cause damage when they combine with active failures to bypass the defence leading to accident causation [25, 26].

## Conclusion

The presence of a retained surgical sponge is considered to be a ‘never event’ by the National Quality Forum of the United States of America and is also part of patient safety guidelines issued by the Health Department of United Kingdom [3]. Shaming the surgeon is not an acceptable solution. It is the collective responsibility of the surgical team, the anaesthetic team, the nursing team and the operating room technicians to ensure the safety of any patient who is brought in through the doors of the operating room.
